# Trans-Boundary Edge Effects in the Western Carpathians: The Influence of Hunting on Large Carnivore Occupancy

**DOI:** 10.1371/journal.pone.0168292

**Published:** 2016-12-21

**Authors:** Miroslav Kutal, Martin Váňa, Josef Suchomel, Guillaume Chapron, José Vicente López-Bao

**Affiliations:** 1 Department of Forest Ecology, Faculty of Forestry and Wood Technology, Mendel University Brno, Brno, Czech Republic; 2 Friends of the Earth Czech Republic, Olomouc branch, Olomouc, Czech Republic; 3 Department of Zoology, Fisheries and Apiculture, Faculty of Agronomy, Mendel University Brno, Brno, Czech Republic; 4 Department of Ecology, Swedish University of Agricultural Sciences (SLU), Riddarhyttan, Sweden; 5 Research Unit of Biodiversity (UO/CSIC/PA), Oviedo University, Mieres, Spain; Sichuan University, CHINA

## Abstract

The conservation and management of wolves *Canis lupus* in the periphery of their distribution is challenging. Edges of wolf distribution are characterized by very few and intermittent occurrences of individuals, which are modulated by multiple factors affecting the overall population such as human-caused mortality, management targets and food availability. The knowledge of population dynamics in the edges becomes crucial when hunting takes place nearby the edges, which may preclude population expansion. Here, using as example the occurrence of wolves in the Beskydy Mountains (Czech-Slovak border), which are the edge distribution of the wolf and Eurasian lynx *Lynx lynx* populations in the West Carpathians, we explored how food availability and hunting in the Slovakian core area affected the dynamics of wolves in the edges of this population. During 2003–2012, we monitored large carnivore occurrence by snow-tracking surveys and tested potential differences in the occurrence of these species in Beskydy Mountains and potential mechanisms behind detected patterns. Despite the proximity to the core area, with several wolf reproductions being confirmed at least in recent years, the wolf was a very rare species in Beskydy and was recorded 14 times less often than the lynx. The expected abundance of wolves in the Beskydy Mountains was inversely related to prey availability in the Slovakian core area. Wolf hunting the year before influenced the expected abundance of wolves in Beskydy area. We discuss how different life histories and legal status of both species probably account for most of the observed difference of occurrence at range margins.

## Introduction

Over the last few decades, we have witnessed a recovery of large carnivores throughout human-dominated Europe [1]. For example, out of the ten wolf *(Canis lupus)* populations currently recognized in the old continent, almost all populations show a stable or increasing trend [1]. Only the wolf population inhabiting Sierra Morena (Spain) shows the opposite pattern, being now at the edge of extirpation [2]. Different factors have been linked to the recovery processes observed for large carnivores in Europe, such as relative political stability following the World War II, the rule of law, favourable conservation legislation, land abandonment together with the recovery of forests and prey populations, and maintenance and revival of traditional livestock management practices [1,3–7].

The processes of recolonization can be slowed down not only by environmental factors at range margins, where populations sometimes approach their ecological limits [8], but also by multiple “*edge effects*” artificially created by humans, such as the outcome of different management strategies implemented across different administrative regions sharing the same population [9] or poaching [10]. For example, the recovery rates of two intensively studied European wolf populations–Scandinavian [11] and Alpine [12] wolf populations–were somewhat lower (λ = 1.04–1.32) than population growth of recovering wolf populations in western Poland (λ = 1.38 [13]), mainly due to a hardly detectable illegal killing of wolves (e.g., up to 51% of total mortality) [12,14].

In expanding populations, large carnivores show vulnerability to various interacting effects at range margins, including hunting and poaching, which may be exacerbated in some recovering areas where people are not used to sharing the landscape with carnivores for a long time, or where the availability of suitable habitat is low [12,14,15]. However, how these factors influence the recovery process of expanding large carnivore populations is still poorly understood beyond fundamental characteristics such as carnivore occupation and abundance. For example, moderate wolf hunting has been suggested to limit the species’ dispersal capabilities through decreased emigration and increased immigration [16,17]. The loss of a dominant individual or the entire breeding pair has been also linked to pack disruption and possible emigration of floaters which can settle down elsewhere [18,19]. On the other hand, wolf dispersal rates can be also modified by higher resource competition caused by lower prey abundance at source populations [20,21]. Since the European landscape, where many wolf populations occur, is intensively used and managed by humans [1,22,23], it can be expected that different factors, such as game hunting (reduction of the wolf prey base), wolf culling and hunting, or habitat fragmentation can also shape the European wolf recolonization process.

The Carpathian Mountains host the second largest wolf population in Europe, with an estimated population size of 3,000 individuals, ranging across 171,200 km^2^ and being shared among 6 countries [1]. Estimates of large carnivores in Slovakia, Ukraine and Romania are not carried out in a standardised manner, and sometimes they are only based on previous year hunts [24]; which is typical in large populations compared to small wolf populations [25]. Wolves in these countries are included in the Annex V of the European Habitats Directive 92/43/EEC and regulated hunting of wolves is allowed. However, in other neighbouring Carpathian countries such as Poland, Czech Republic or Hungary wolves are strictly protected by national laws and, except of Poland, also listed in Annex IV of the Habitats Directive. Therefore, the same wolf population is shared by different countries where wolves have different legal status and management strategies, and management goals are different, which is expected to translate into trans-boundary edge effects affecting wolf dynamics at different scales. Implications of the existence of such edge effects are substantial because they may compromise management and conservation goals and obligations at the Member State level. For example, the conservation status of wolves in the Czech Republic is classified as “bad” according the EU report under Article 11 of Habitats Directive [26] and the wolf recovery from the east depends on the dynamic of the species in Slovakia and Poland.

Here, using wolves in the West Carpathians as a case study, we explored how wolf hunting and prey availability (modulated by game hunting) in the Slovakian wolf core area influenced the wolf recovery process at the population edge of the Czech-Slovakian borderline, namely the Beskydy area. We compared this pattern with data for Eurasian lynx *(Lynx lynx)* in the same area, which is fully protected in both countries and was used to explore the influence of protected status on trans-boundary edge effects. Our null hypothesis was the lack of influence of hunting in the Slovakian core area on the occurrence and re-colonization dynamics of wolves in Beskydy area (e.g., no effect of hunting, both for wolves and wild prey, on wolf dynamics in Beskydy area). Alternatively, and not necessarily mutually exclusively, we tested whether i) hunting limits wolf dispersion from the Slovakian core to Beskydy (expected to be an attractive sink considering prey abundance and habitat, see below), decreasing the number of dispersers reaching this area [16,17]; ii) hunting disrupts social organization and the structure of wolf packs, increasing the number of lonely wolves and floaters [18,19]; and iii) dynamics of prey availability in the Slovakian core area–modulated by game hunting—influences the probability of wolf occurrence and expected abundance in Beskydy. The number of wolves detected in Beskydy area would be thus determined by variation in prey abundance in the Slovakian core, which is expected to impact and restructure the social organization of wolves, potentially affecting the number of dispersal and floaters [20,21].

## Materials and Methods

### Study area

**Fig 1:**
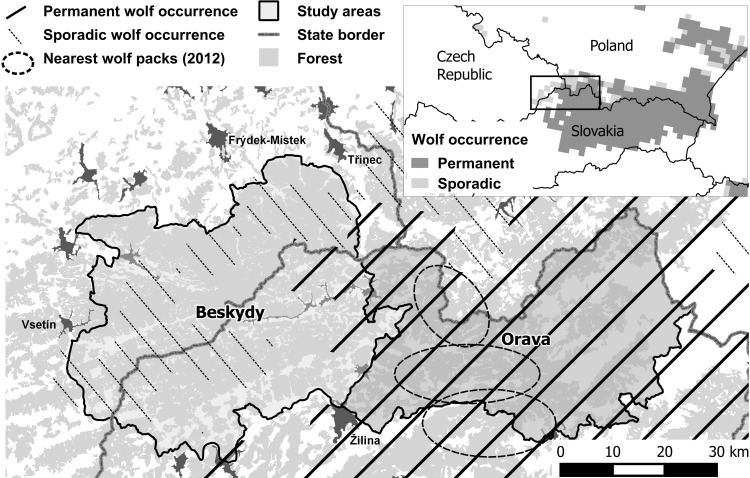
Beskydy and Orava study areas in the West Carpathians, Czech and Slovak Republics, representing the *periphery* (Beskydy)–sporadic occurrence—and *core* population (Orava) in the area in 2011, according to Chapron et al [1]. The approximate locations of the wolf packs nearest the periphery were mapped during this study in 2012 (see methods for details).

The West Carpathian Mountains are situated at the Czech-Slovak-Polish borderlands, at the western edge of continuous wolf and lynx occurrence in the Carpathians [1] (Fig 1). Our study area, “Beskydy” (49°10’–49°39’; 17°59’–18°44’) represents a periphery of large carnivore occurrence. This large forested area is in the Czech-Slovak borderlands designated as Protected Landscape Area (PLA) Beskydy (on the Czech side: 1,160 km^2^) and the south part of PLA Kysuce (on Slovak side: 428 km^2^). Altitude ranges from 350 to 1,324 m a.s.l. and snow cover persists for 120–180 days with respect to the altitude and the direction of the slope [27,28].

Forest covers most of the landscape (72%). The stands dominated by Norway spruce (*Pica abies*) and beech (*Fagus sylvatica*) are intensively managed and vast areas have been converted into forest plantations–only a small proportion of natural forests (2.6%) are protected in nature reserves. The average human density is approximately 100 inhabitants per km^2^ (on the Czech side of the border), with most people being concentrated in towns and villages in valleys and basins. However, many activities are situated up in the mountains. Besides forestry and hunting practices, there is a moderate impact of tourism, reaching up to 65,000 visitors per year in different parts of Beskydy Mts. [29]. There are also number of ski lifts, ski and bike routes and hiking paths, recreational cabins and chalets.

The Slovakian side is expected to act as the source of wolves reaching the Beskydy area. We used the so called “*hunting districts*” in Slovakia, the smallest units where numbers of killed ungulates and wolves were available (on average 697±150 km^2^) for this study. We joined Beskydy PLA (on the Czech side) with two adjacent hunting districts in Slovakia (Javorníky I and II) and created “*periphery*” (i.e. Beskydy area), since the permanent distribution of wolves known in between the Czech Republic and Slovakia is mostly situated out of this region, and no stable packs were known from this area during the last years (Fig 1). The Slovakian core area of wolf distribution, namely *“Orava”*, consisted of two neighbouring hunting districts (Slovenské Beskydy and Oravská Magura), which together represent the area with the permanent wolf distribution in the region [1]. We called this second area the “*core*” population. Basic environmental characteristics of the periphery and core areas are described in Table 1. Prey biomass and forest cover were higher in the *periphery* (Table 1).

**Table 1:** The basic environmental characteristics (forest cover and road density), and average prey biomass, wolf hunting and wolf abundances reported by hunters in the Slovakian core area (Orava) and the periphery (Beskydy) between 2003 and 2012.

Study area	Area (km^2^)	Forest cover (%)	Road density (km/km^2^)	Average annual prey biomass (kg/100 ha)	Wolf annual hunting	Reported wolf annual abundance (ind./100 km^2^)
Orava	2,329	50.2	0.30	182.1	91	6.9
Beskydy	1,660	68.3	0.30	307.1	0	0.7

Forest cover was higher in Beskydy compared to Orava (Z-proportions test = 11.30, P < 0.001).

### Occurrence of large carnivores in Beskydy area

Once wiped out from the area which today is the Czech Republic, a combination of legislative protection in Slovakia and socioeconomic changes in countryside caused the natural return of lynx in the Beskydy area in the latter half of the 20^th^ century [30]. Lynx were again almost exterminated in Beskydy in early 1970s after a period of legal hunting, and following a subsequent hunting ban, the population recovered once more [31], reaching 11 independent individuals in 2011–2013 [32] with about 1–3 reproductions per year [33]. The two periods of lynx recovery are connected with the partial lynx protection in Slovakia between 1936 and 1955, and after 1975, increasing the distribution range in following decades [34]. The lynx is a fully protected species in Slovakia since 1999 with the estimated lynx population reaching 300–400 individuals [1].

Wolves were exterminated from the West Carpathians in the beginning of the 20^th^ century in the Czech Republic [35] and in the north-west area of Slovakia [36]. Afterwards, wolves reappeared in the 1940’s in Orava region in west Slovakia (i.e. Slovakian core) [36] and more records of shot wolves from north-west Slovakia and Beskydy are available from 1965–1968, but the area of continuous wolf occurrence was located in east Slovakia [37]. In early 1970s, the number of killed wolves decreased because of overhunting in previous years and the distribution shrunk even more to the east [37]. The current permanent wolf occurrence in central and north-west Slovakia (Fig 1) started in the beginning of the 1980s [38], but the wolf remains on the list of hunting species up to now. Currently there is a partly open hunting season and between 74 and 159 (on average 115.7±24.8) animals have been legally killed each year in the study period 2002/2003–2011/2012 across Slovakia [39]. The estimated wolf population in Slovakia is 200–400 individuals [1]. During the study period, the number of killed wolves in the Slovakian core area varied between 2 and 17 wolves per year (on average 9.1±4.1) and the number did not change significantly over time (r_s_ = 0.256; p = 0.475). Number of packs or reproducing pairs was not known for the whole core area. Between 2011 and 2012 we confirmed 3 packs in the Slovakian core area at the distance 10–50 km from the Czech-Slovakian border, a centre of Beskydy area. These findings are complementary to Nowak et al. [40], where 3 packs on the Polish side of Orava region were detected during the years 1998–2003. The same territory was confirmed to be occupied in our study in 2003–2012 (S. Nowak; pers. comm.). This information suggests that the border of permanent wolf occurrence did not change during the study period.

Wolves were first recorded in the Czech Carpathians–Beskydy–in 1994 [41] and 1–3 wolf packs were registered in following years until 2003 [42]. Anděra and Červený [35] considered wolf occurrence in Beskydy after 2003 stable and even higher, but these were only estimates, not based on verified and reliable data. The current status of the species was verified during our study.

### Data collection

Between 2003 and 2012, we monitored wolf and lynx occurrence in Beskydy area (ca. 1,660 km^2^) by snow-tracking surveys, where footprints and other signs of carnivore occurrence (e.g., scats) were registered. Because of the mountainous habitat with incessantly changing conditions for snow-tracking and demanding character of winter monitoring, no fixed transects were set. We aimed to cover an area as large as possible by organizing regular 2–3 days long monitoring sessions taking place almost every weekend in December–March each year. Experts and trained volunteers covered on foot, snowshoes or cross-country ski, in small groups with at least one experienced person or individually, 10–35 km long trails. During the study period, a total of 2,177 walked trails were recorded using hand GPS devices or marked in the copies of maps and later georeferenced in GIS software. All lynx and wolf tracks crossing the walked trails were recorded and followed, where possible, in order to determine the number of individuals travelling together, direction and other signs. In the Slovakian core (ca. 2,329 km^2^), on the other hand, we carried out a specific monitoring in order to confirm wolf reproduction and the approximate location of packs ranging near the Beskydy area (Fig 1). Such monitoring was only carried out between 2011 and 2012, with 18 snow-tracking surveys made in winters of 2010/2011 and 2011/2012. In addition, reproduction was also confirmed in summer 2012 by searching for places of pup rearing and simulated howling [40,43]. Such monitoring was carried out also during summer in the edge area, but with no success. Since the field research was non-invasive and was not conducted in areas with limited access, no official permit was needed according to the national legislative.

Data on wolf and ungulate hunting in Slovakian hunting districts were requested from National Forest Centre, the Slovakian authority responsible for hunting statistics. We also gathered information on ungulate hunting in the Czech section of Beskydy Mountains (PLA Beskydy) from 9 regional authorities in order to compare food availability between areas. In both countries, hunters have to report the bags of all game species to the district authorities by the end of the “*hunting year*” (i.e. 31st March; e.g. data from year 2003 were recorded from April 2002 to March 2003). This system complements very well the data collected during the winter seasons through large carnivore monitoring, as the continuous snow cover usually ends in March. Hunting data are a commonly used source of ungulate indices at large spatial scales and for long periods where other sources are not available (e.g. [44–46]). Given the similar social conditions and the use of same hunting system across the study area, we ruled out different intensity of hunting efforts as a possible reason for significant differences in ungulate density and total biomass in Beskydy and Orava. The total ungulate densities (0.8–1.3 animals/km^2^ for red deer *Cervus elaphus*, 0.9–2.5 for roe deer *Capreolus capreolus* and 1.4–6.8 for wild boar *Sus scrofa*) fall into the lower or middle range reported from other areas in Europe [47–49].

### Data analysis

Carnivore data were classified according to the SCALP (Status and Conservation of Alpine Lynx Populations) methodology used in Swiss Alps for lynx [50]. This classification consists of 3 categories of reliability for field information reporting large carnivore occurrence: (1) hard evidence, i.e., direct signs of species presence (photo, dead animal, genetic proof), (2) non-direct signs, especially tracks, scat, remains of prey, confirmed by experienced observers and (3) non-verified signs reported by public or direct observations without documentation [50]. Because wolf footprints can easily be confused with those of dogs, especially in areas with low wolf density and high human activities [51], we added the following criterion for wolf tracks in the second category of SCALP reliability: the tracks must be followed at least 500 m without the presence of human tracks in order to assess wolf behaviour, find a scat or distinguish the number of animals, similar to German monitoring standards [52]. In this study, only reliable data from categories 1 and 2 were used for subsequent analyses.

The area occupied by wolf and lynx in Beskydy over time was evaluated at the level of 10 × 10 km EEA cells (n = 12). A cell was considered as monitored in this study if every year during the study period a transect crossed the cell for at least 2 km. When several trails were carried out in the same cell and on the same day, transects were merged and they were considered as one visit. Moreover, trails walked during bad weather–snowing, windy weather or other adverse conditions–and associated information were excluded for subsequent analyses. A total of 15,908 km surveyed at the 10 × 10 km cell level were considered in this study. Due to voluntary-based work and increasing interest of people in monitoring, the number of trail sections increased over time (r_s_ = 0.94; P < 0.001). A cell was considered as positive in terms of wolf/lynx occurrence in case a track of a large carnivore was detected during a visit regardless how many times a track was found on the same day. Finally, a dataset of negative and positive visits (binary coded) in each cell was arranged for subsequent analyses.

Sparse data from spatially replicated count surveys can be utilized effectively for estimation of population abundance using N-mixture models [53]. We estimated the expected abundance of wolves in Beskydy from repeated counts using the “*unmarked”* package [54] for R software [55]. We built and compared eight competing models considering the influence of different combinations of the following predictors measured at the Slovakia core area on the expected number of wolves occurring in the Beskydy area during our study period: wolf hunting, wolf hunting the year before (to explore for time-delayed effects in dispersal associated to disruption in wolf packs) and prey biomass. We compared: i) a null model; ii) a full model; iii), iv) and v) models considering each predictor separately; vi) a model considering synergic effects of hunting (hunting the year before + hunting); vii) a model considering the combination of hunting the year before and prey biomass; and finally, viii) a model considering the combination of hunting and prey biomass the same year. All variables were scaled before analyses. We also tested for the best data distribution considering the structure of our dataset (Poisson, Negative Binomial and Zero-inflated Poisson models) and found that Poisson was the best error distribution for our analyses (based on Akaike Information Criterion values). The covariate affecting wolf detectability *“p”*, i.e. transect length (km), was considered in all models. Akaike Information Criterion (AIC) was used for model selection [56]. Models within ΔAIC <2 were considered to have substantial empirical support [56]. We also used the AIC weights (*w*_*i*_) to determine the relative strength of support for each competing [56]. All statistical analyses were performed in R 3.0.2 [55].

Since the official deer and wild boar abundance reported by hunters was several times underestimated in Czech and Slovak conditions, we used a reverse calculation method [57] in order to get more reliable figures on abundance and prey biomass Details on this calculation are described in S1 File. Estimated abundance and biomass of all ungulates was significantly higher in the periphery (Beskydy area) compared to the core (Wilcoxon paired test Z = 2.80 P = 0.0051; Table 2).

**Table 2:** Estimated prey (ungulate) abundance and biomass per 100 ha (average values and ranges are showed).

	Roe deer	Red deer	Wild boar	Average total biomass (kg)
Slovakian Core (Orava)	0.95*	0.67*	2.42*	182.06*
	(0.82–1.16)	(0.54–0.87)	(1.38–3.84)	(126–262)
Periphery (Beskydy)	2.26	0.99*	4.14*	307.12*
	(1.85–2.53)	(0.82–1.26)	(2.17–7.21)	(214–447)

* Asterisks denote statistically significant increase during years 2003–2012 (based on Spearman correlation analyses).

## Results

**Fig 2:**
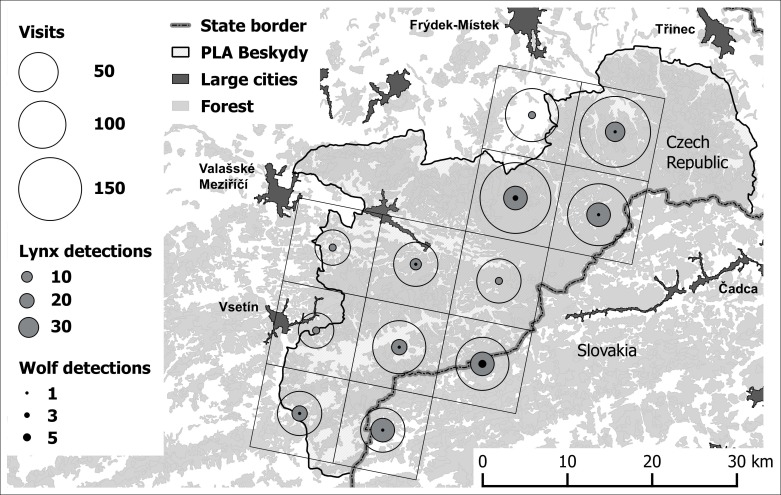
The Beskydy area with the sampled cells. The size of open circles represents the number of visits in each cell, grey and black inner parts represent number of lynx and wolf detections respectively.

Even though Beskydy was close to the Slovakian core area, with several wolf reproductions being confirmed at least in recent years (Fig 1), wolves were very rarely detected in the Beskydy area during the study period (note that distances between the core and any part of the Beskydy area are less than 100 km). Overall, over the ten-year study period, wolves were detected only in 16 visits out of the 1,264 visits carried out during the study period (1.27%) (Table 3). The estimated wolf range (sporadic) and the number of wolf detections did not increase over time (r_s_ = -0.22; P = 0.541), although the number of visits had a 3-fold increment (Table 3). Lynx, on the contrary, were detected, on average, 14 times more often than wolves in the same area, with these differences being significant (Fig 2; t = -4.49; P = 0.001). The number of lynx signs increased during the 10-year period (r_s_ = 0.89; P = 0.006). But this increase was mainly related to the increase in the number of visits over time (r_s_ = 0.88; P = 0.001), since the occupied area remained approximately constant during the study period (Table 3; r_s_ = 0.32; P = 0.371).

**Table 3:** Summary of the monitoring results by year.

Season	2002/03	2003/04	2004/05	2005/06	2006/07	2007/08	2008/09	2009/10	2010/11	2011/12
Visits	55	83	127	94	94	134	168	155	179	175
Lynx detections	10	11	20	12	12	22	14	39	34	53
Lynx EDR (km^2^)	600	600	700	800	500	900	700	700	600	800
Wolf detections	1	1	3	1	1	4	2	3	0	0
Wolf EDR (km^2^)	100	100	200	100	100	400	200	300	0	0

Estimated Distribution Range (EDR) is based on number of 10 × 10 km EEA with confirmed species presence.

The dynamics of wolves in Beskydy (i.e., the expected number of wolves occurring in the periphery area) was modulated by prey biomass and wolf hunting in the core area (models ΔAIC <2) (Table 4). The most parsimonious model was the model considering prey biomass in the core area (*w*_*i*_ = 0.33) (Table 4). The expected abundance of wolves in the Beskydy area was significantly and inversely correlated with prey biomass in the Orava area (Table 5). Three additional models showed ΔAIC <2 (Table 4): the models considering the combination of wolf hunting the year before and prey biomass (*w*_*i*_ = 0.24), wolf hunting the year before (*w*_*i*_ = 0.14), and the combination of hunting the same year and prey biomass (*w*_*i*_ = 0.12) (Table 4). From these predictors, wolf hunting the year before significantly and positively influenced the expected abundance of wolves in Beskydy area (S2 Table).

**Table 4:** Comparison of the eight competing models built to explore how the dynamics of wolves in the Beskydy area was influenced by prey and wolf hunting in the core (Orava area).

*Competing models*	*AIC*	*ΔAIC*	*w*_*i*_
lam(prey biomass)p(km)	166.87	0	0.33
lam(hunting year before + prey biomass)p(km)	167.46	0.59	0.24
lam(hunting year before)p(km)	168.59	1.72	0.14
lam(hunting + prey biomass)p(km)	168.86	1.99	0.12
lam(hunting year before + hunting + prey biomass)p(km)	169.46	2.59	0.09
lam(hunting year before + hunting)p(km)	170.59	3.72	0.05
lam(hunting)p(km)	172.09	5.21	0.02
lam(.)p(.)	174.90	8.03	0.01

Models are ranked from the best candidate model (lowest AIC value).

**Table 5:** Parameter estimates (± SE) for the best candidate model explaining the dynamics of wolves in the Beskydy area (periphery) in relation to prey biomass in the core area.

*Parametric coefficients*	Estimate	SE	*P*
***Abundance***			
Intercept	3.43	1.19	
Prey biomass	-0.63	0.28	0.026
***Detection***			
Intercept	-8.46	1.23	
Transect length (km)	0.04	0.01	<0.001

## Discussion

In Beskydy area, between 2003 and 2012, wolf detections were very rare and no pack formation was documented, suggesting only occasional wolf visits in the area. For example, three times higher monitoring effort in 2010 resulted in a similar number of wolf signs than in 2003, and no reliable wolf visits have been detected since the winter of 2010/11 (Table 3). The sporadic occurrence of wolves in Beskydy was obvious also from the data reported by hunters. Population abundance estimated by hunters in Beskydy was almost ten times lower than in Orava region, according to hunters’ reports (Table 1). In addition, the amount of claimed wolf damages on sheep were low in Beskydy (16.3±14.5 sheep/year) showing a negative trend over time [7].

The dynamic of wolves in the periphery (Beskydy) was mainly modulated by variations in ungulate prey biomass in the Orava core area. These findings suggest that shortage of food in the core may trigger wolf dispersion to the periphery. Increased resource competition in saturated wolf populations has been linked to increments in wolf dispersal rates [20,21]. Elsewhere, dispersal rates of recovering population in Central Rocky Mountains slightly, but non-significantly, declined during times of lower ungulate densities [58]. The West Carpathian population did not expand west during the study period, thus the observed pattern was similar to saturated populations. The amount of dispersals at the periphery was also positively influenced by wolf hunting in the Slovakian core area the year before. According to the review by Bainerd et al [19], wolf packs dissolved and abandoned their territories when a breeder was killed in 38% cases, resulting also in smaller group sizes. Nowak et al. [40] found that the packs along Polish-Slovakian border in years 1998–2003 were smaller and did not increased in contrast to the packs situated inland, far from area of wolf killing, which increased during the study period. Spatially heterogeneous hunting pressure (year-round protection in Poland versus open hunting season in Slovakia) may have induced a source–sink wolf dynamics in this border ([59]; see also [60]). Hunting located in high quality habitats may create “*attractive sinks”* [61,62]. Roughly, 40% of Slovakian wolf packs had territories that spanned an international border according to the census in 2005/2006, predominantly with Poland [63], thus patches with different hunting intensity can occur along the border.

Assuming that wolf densities in Orava area were around 1.7–2.5 wolves/100 km^2^, i.e. the same as at the beginning of the study period [40], the average number of wolves legally killed in the Slovakian core (on average 9.1 individuals/year) accounted for hunting of 30–44% of an estimated wolf population in Orava region.

Which factors, other than hunting, could explain different abundance of wolves and lynx in the periphery of their occurrence in the West Carpathians? Firstly, we exclude prey availability, which was 85% higher in the periphery than in the Orava core area. Secondly, habitat differences can hardly explain the observed patterns in both species. Lynx is more sensitive to forest fragmentation and transport infrastructure than wolves [64–66]. Since the forest cover was higher in the periphery than in the core area and road density was similar (Table 1), we would expect less lynx than wolves in view of the habitat available. Thirdly, we considered undetectable (cryptic) poaching of wolves [14] or different level of poaching on both species. While the wolf is a game species with hunting quotas in Slovakia, its protected status may not be acknowledged by Czech hunters living in a culturally similar environment, which may lead to higher level of illegal hunting–than in the case of lynx. However, we know the lynx is also illegally hunted in the Czech Republic despite its year–round protection in both countries [67] and due to the absence of comparable and reliable data on the extent of poaching for both species here, we cannot judge this argument properly. Fourthly, different patterns and strategies of recolonization occur with wolves. Dispersers can settled down in a neighbouring territory of their maternal pack [68] or cross large areas of suitable habitat before settling [11,69]. Reduced dispersal to Beskydy could be attributed to conspecific dispersal patterns [70], where wolves preferably dispersed to areas of high wolf densities [58]. Due to potential Allee effects, reduced probability of finding mates at low densities further slows the predicted rate of recolonization [71]. Interestingly, most of wolf detections in Beskydy did not occur in the same cell during the study period. A female-wolf was hit by a car in 2012 at the very western edge of Beskydy area, probably after some time the animal had spent in large forested patches in Beskydy [72], suggesting a wolf was only passing through the area rather than settling. On the contrary, instead of long-distance dispersal through human-dominated landscape typical of wolves [73,74], lynx prefer to settle down in neighbouring vacant territories [75]. The lynx population in Besykydy has been stable since the 1980s [33,35] and the species is fully protected in Slovakia. In summary, both life history and legal status of species are probably accounted for in the observed difference of occurrence between these two species.

## Conclusions

For those carnivore populations listed in Annex V of the EU Habitats Directive and with annual hunting quotas, sustainable management and robust monitoring is an essential requirement [76], as well as the integration of undesirable side effects of hunting in large carnivore strategies [77]. Chapron et al. [78] proposed an adaptive management strategy for sustainable management of wolves removing a moderate percentage (10%) of the population, whenever the population has grown by more than 5% in the previous year. Such a recommendation is hard to apply in the absence of reliable and long-term monitoring, but estimated wolf mortality in our case (roughly 30–44% of Orava population) fairly exceeds this conservative limit. Although Slovakia as an EU Member State is obliged to monitor the status of the species listed in the Habitats Directive, the species’ status is assessed mostly on the basis of expert estimates without reliable monitoring and knowledge of basic population parameters. A pilot study in the central Slovakia [79] demonstrated the current lack of capacity for science-based population management.

Achieving a favourable conservation status (FCS) [80] for wolves in the Czech Republic depends on wildlife management in Slovakia. Since Member States have individual obligations to promote FCS within their borders [80] a transboundary approach to the management of large carnivores at the population level is necessary [9,76]. Despite the framework provided by the Bern Convention and guidelines available within European Union, conservation and management rarely occurs at an international level [5]. Large carnivores with large home ranges or territories are particularly affected by different management approaches across European borders, but the trans-boundary management at the population level and related legal issues pose challenges for conservation of many other species in different parts of the world [81].

Additionally, killing terrestrial animals has been recognized as one of the most significant threat in a Carpathian transboundary protected area spreading across Poland, Slovakia, and Ukraine [82]. A new management plan for wolves in Slovakia has been recently approved and contains more strict rules in terms of killing wolves, e.g. a ban of wolf hunting in Natura 2000 sites where the species is a subject of protection and within the buffer zone neighbouring with Poland and partly also along the Czech-Hungarian border [83]. Scientifically-sound monitoring approaches are urgently needed in order to set sustainable management of wolves. In regions where monitoring possibilities are limited, securing large and regularly distributed source areas for hunted species may be more effective than trying to regulate quota size [59]. Time will show if these areas in Slovakia are large enough and could lead to wolf recovery at range margins.

## Supporting Information

S1 File Calculation of estimated ungulate abundance and biomass. (PDF)

S1 Table Comparison of the eight competing models built to explore how the dynamics of wolves in the Beskydy area was influenced by prey and wolf hunting in the Orava core area using hunted ungulate biomass (kg/100 ha). Models are ranked from the best candidate model (lowest AIC value). N = number of parameters
(PDF)

S2 Table Parameter estimates (± SE) for the rest of models explaining the dynamics of wolves in the Beskydy area and showing ΔAIC <2. (PDF)
