# Supplementary material for: Trans-Boundary Edge Effects in the Western Carpathians: The Influence of Hunting on Large Carnivore Occupancy
Source: PLoS One. 2016 Dec 21;11(12):e0168292. doi: 10.1371/journal.pone.0168292 (PMC5176292; doi:10.1371/journal.pone.0168292)
Supplement: S1 File — (PDF) [file pone.0168292.s001.pdf]

S1 File. Calculation of estimated ungulate abundance and biomass

The reverse calculation method (Plhal & Kamler, 2012) was used to estimate abundance of prey in each area by using an expected reproduction rate (RR) for each species and the expected sex ratio of the population. We used the following values of RR for roe deer, red deer and wild boar, respectively: 0.8, 0.75 and 3.2 (figures extracted from the Regulation of Czech Ministry of Agriculture; No. 491/2002 Coll. about the creation of hunting plans). Such values were selected at the lower end of the available range for each species, taking in account a lower productivity in mountain forests. Next, considering the total number of hunted individuals in each area, the number of adult females needed to get the annual population increase was calculated using the formula: number of females = new-borns/RR. Finally, the total population size at the end of the hunting season was derived from the ratio between males, females and fawns (we assumed average values according to Plhal & Kamler (2012), and also values listed in the abovementioned hunting regulation (40% of females, 40% of males and 20% of fawns). The total amount of prey (ungulate) biomass potentially available in each area was calculated according to Nowak et al. (2005): 19 kg for roe deer, 100 kg for red deer and 40 kg for wild boar. The reverse calculation method suppose fixed reproductive rate and that the average number of hunted individuals was equal to new-borns. This hypothesis is, however, true only in ideal conditions when the number of new-borns equals the number of hunted animals every year, what is hard to test in absence of data about real new-borns. Thus, we used also data of real harvested biomass without recalculation to supposed available biomass and ran the analysis (S1 Table) separately with these values to be sure our results were not influenced by an imprecise conversion. However, because all the trends and values were almost the same we present the results with the estimated prey biomass in the main text.

References:

Nowak, S., Mysłajek, R. W., & Jędrzejewska, B. (2005). Patterns of wolf Canis lupus predation on wild and domestic ungulates in the Western Carpathian Mountains (S Poland). *Acta Theriologica*, *50*(2), 263–276.

Plhal, R., & Kamler, J. (2012). Analysis of accuracy of hunting plan in the Czech Republic. *Acta Universitatis Agriculturae et Silviculturae Mendelianae Brunensis*, *LX*(3), 165–172.
