# Supplementary material for: Trans-Boundary Edge Effects in the Western Carpathians: The Influence of Hunting on Large Carnivore Occupancy
Source: PLoS One. 2016 Dec 21;11(12):e0168292. doi: 10.1371/journal.pone.0168292 (PMC5176292; doi:10.1371/journal.pone.0168292)
Supplement: S1 Table — Models are ranked from the best candidate model (lowest AIC value). N = number of parameters (PDF) [file pone.0168292.s002.pdf]

**S1 Table. Comparison of the eight competing models built to explore how the dynamics of wolves in the Beskydy area was influenced by prey and wolf hunting in the Slovakian wolf source, using hunted ungulate biomass (kg/100 ha). Models are ranked from the best candidate model (lowest AIC value). N = number of parameters**

| MODEL | N Parameters | AIC | deltaAIC | AICweight |
|---|---|---|---|---|
| lam(prey biomass)p(km) | 4 | 166.31 | | 0.36 |
| lam(hunting year before + prey biomass)p(km) | 5 | 167.13 | 0.82 | 0.24 |
| lam(hunting + prey biomass)p(km) | 5 | 168.29 | 1.99 | 0.13 |
| lam(hunting year before)p(km) | 4 | 168.59 | 2.29 | 0.11 |
| lam(hunting year before + hunting + prey biomass)p(km) | 6 | 169.12 | 2.81 | 0.09 |
| lam(hunting year before + hunting)p(km) | 5 | 170.59 | 4.28 | 0.04 |
| lam(hunting)p(km) | 4 | 172.09 | 5.78 | 0.02 |
| lam(.)p(.) | 2 | 174.90 | 8.59 | 0.01 |
