# Supplementary material for: Trans-Boundary Edge Effects in the Western Carpathians: The Influence of Hunting on Large Carnivore Occupancy
Source: PLoS One. 2016 Dec 21;11(12):e0168292. doi: 10.1371/journal.pone.0168292 (PMC5176292; doi:10.1371/journal.pone.0168292)
Supplement: S2 Table — (PDF) [file pone.0168292.s003.pdf]

**Table S2. Parameter estimates (± SE) for the rest of models explaining the dynamics of wolves in the Beskydy area and showing ΔAIC <2. Model considering hunting year before and prey biomass:**

| ***Parametric coefficients*** | **Estimate** | | ***P*** |
|---|---|---|---|
| ***Abundance*** | | | |
| Intercept | 2.96 | 1.12 | |
| Prey biomass | -0.53 | 0.29 | 0.077 |
| Hunting year before | 0.30 | 0.25 | 0.232 |
| ***Detection*** | | | |
| Intercept | -7.95 | 1.16 | |
| Transect length (km) | 0.04 | 0.01 | <0.001 |

**Model considering hunting and prey biomass:**

| ***Parametric coefficients*** | **Estimate** | | ***P*** |
|---|---|---|---|
| ***Abundance*** | | | |
| Intercept | 3.43 | 1.19 | |
| Prey biomass | -0.63 | 0.29 | 0.028 |
| Hunting year before | 0.02 | 0.26 | 0.932 |
| ***Detection*** | | | |
| Intercept | -8.46 | 1.23 | |
| Transect length (km) | 0.04 | 0.01 | <0.001 |

**Model considering hunting the year before**

| ***Parametric coefficients*** | **Estimate** | **SE** | ***P*** |
|---|---|---|---|
| ***Abundance*** | | | |
| Intercept | 1.25 | 3.24 | |
| Hunting year before | 0.50 | 0.25 | 0.050 |
| ***Detection*** | | | |
| Intercept | -6.27 | 3.27 | |
| Transect length (km) | 0.04 | 0.01 | 0.003 |
